# Agile Nudge University Innovation Forum: solving real-world problems

**DOI:** 10.3389/fpubh.2025.1585562

**Published:** 2025-09-22

**Authors:** Cristina Barboi, Fatemehalsadat Shojaei, Leslie Gardner, Nick Stewart, Fereshtehossadat Shojaei, Mark Seidman, Richard J. Holden, Nicole R. Fowler, Diana Summanwar, Brooke Stephanian, Malaz Boustani

**Affiliations:** ^1^School of Medicine, Indiana University, Indianapolis, IN, United States; ^2^Department of Anesthesiology, School of Medicine, Indiana University, Indianapolis, IN, United States; ^3^Center for Health Innovation and Implementation Science, School of Medicine, Indiana University, Indianapolis, IN, United States; ^4^Luddy School of Informatics, Computing, and Engineering, Indiana University Bloomington, Bloomington, IN, United States; ^5^Center for Aging Research, Regenstrief Institute, Inc., Indianapolis, IN, United States; ^6^Department of Health and Wellness Design, School of Public Health, Indiana University Bloomington, Bloomington, IN, United States; ^7^Sandra Eskenazi Center for Brain Care Innovation, Eskenazi Health, Indianapolis, IN, United States

**Keywords:** community platforms, agile science, nudges, innovation forums, healthcare solutions

## Abstract

**Introduction:**

The Indiana University Innovation Forum is an online group-based problem-solving platform that brings together physicians, nurses, social workers, patients, healthcare managers, and other key stakeholders to tackle complex healthcare challenges. This study analyzes the data generated during the Agile Nudge University Innovation Forum (ANUIF) events from October 2022 to December 2024.

**Methods:**

This is a mixed-methods study consisting of a quantitative analysis of the ANUIF events, including participant numbers, level of satisfaction, and the number of solutions created, as well as a qualitative appraisal of the solution themes generated by participants.

**Results:**

The average number of attendees for the Innovation Forums was 14.83 (SD = 6.92), and the mean satisfaction score was 51.56 (SD = 25.08). The average number of generated solutions per forum was 16 (SD = 5.1), with an average of 10.71 (SD = 3.16) *main solutions and* 4.38 (SD = 3.71) *sub-solutions.* The average number of old or *existing solutions* was 6.19 (SD = 2.94), and the average number of *novel solutions* was 4.52 (SD = 2.44). The administrative changes, followed by the implementation of the ANUIF dashboard and control charts, resulted in significant increases in the number of participants contacted, respondents, registered attendees, and actual attendees. There were significant differences in the average Net Promoter Scores between the attendee cohorts included in the study. The qualitative analysis of Innovation Forums identified five common themes; the most common themes were *Adaptive innovation* and *Collaborative Problem-Solving*.

**Discussion:**

A streamlined Innovation Forum process ensured a steady number of participants with average satisfaction scores. The attendees generated innovative, generalizable solutions applicable to “real-world” healthcare challenges. Participants generated more than one solution and sub-solutions to the discussed problems, demonstrating an understanding of agile science-based problem-solving, ideation, and innovation.

## 1 Introduction

Online community platforms, such as PatientsLikeMe and Mayo Clinic Connect, allow individuals to shared experiences and knowledge across geographical boundaries (1–4). These platforms harness diverse perspectives to address challenges across various industries, including healthcare (5–8). In healthcare, online, virtual communities engage patients and providers, support education, enable simulation and knowledge sharing, and promote research and innovation (9). Despite the development of online health communities, there is minimal empirical research on how to establish and sustain them for long-term, solution-oriented engagement (10–12). In recent years, there has been a notable increase in digital platforms that connect patients, caregivers, and medical professionals to share experiences, knowledge, and support (13–15).

Online Innovation Forums are a private community platform for collaborative problem-solving sessions that bring together physicians, nurses, social workers, patients, healthcare managers, and other key stakeholders to tackle complex healthcare challenges. In 2021, Indiana University’s innovative Agile Nudge University (ANU) program launched the Innovation Forum (IF) online group-based problem-solving platform, which was funded by the NIH’s National Institute on Aging. The online curriculum offers interactive learning through virtual meetings and assigned readings (16).

The Innovation Forum (IF) was conceptualized as a virtual innovation community designed to generate behavioral nudges for healthcare improvement. Grounded in virtual community theory (17, 18), virtual innovation communities leverage digitally mediated networks to accelerate knowledge sharing and collaborative problem-solving. During the IF, healthcare providers and patients often develop practical, experience-based insights that can be shaped into effective nudges. By applying principles of open innovation, ANUIF facilitates cross-boundary idea generation and refinement, positioning itself as a digital laboratory for co-created solutions. Its iterative cycles of ideation and feedback, rooted in the Agile Science model (19, 20), emphasize rapid experimentation, adaptive learning, and continuous improvement. Through this integration, the IF functions as a digitally enabled innovation ecosystem, capable of transforming dispersed experiential knowledge into scalable, evidence-informed strategies that accelerate the design and diffusion of context-specific nudges across healthcare systems.

Unlike traditional forums that may focus solely on discussion, the Agile Nudge University Innovation Forum (ANUIF) applies Agile Innovation principles to drive actionable, evidence-based behavioral strategies. This approach emphasizes rapid experimentation and iterative problem-solving, enabling the swift translation of innovative ideas into practice (18). The ANUIF is a monthly event that empowers and facilitates the design, development, and application of evidence-based behavioral strategies, or “nudges,” intended for solving real-world problems or “challenges” encountered in research and clinical practice. “Nudges,” according to Thaler and Sunstein, are small adjustments made to the architecture of choices in order to affect behavior. Nudges have been demonstrated to enhance decision-making and compliance in the healthcare industry (21–23). These concepts are applied by ANUIF through an agile innovation framework that facilitates quick iterations and practical implementation (24, 25). In healthcare, “nudges” facilitate the rapid innovation and implementation of new standards of care based on scientific evidence (26, 27). The agile science principles serve as the foundation for developing “nudges” by incorporating iterative and incremental approaches, feedback loops, and continuous improvement (28). Therefore, “nudges” are a flexible, rapidly evolving method for discovering and applying knowledge in a dynamic, real-world setting. Using the insights from agile science, the ANUIF requires strict adherence to a minimally specified process to ensure maximum participant engagement and yield minimally viable solutions for those involved (19). When launched, the benchmarks for a successful ANUIF were set at a minimum attendance count of at least 10 participants for each monthly event. Despite significant investments by healthcare systems and research organizations in solutions to improve healthcare, it remains challenging to build communities that consistently foster scalable and implementable innovations (9). The ANUIF tackles two common issues that arise when creating virtual communities. First, by hiring people from a wide range of positions and skills, the ANUIF preserves group cohesion while maintaining a varied viewpoint by emphasizing Agile Science and Nudge (29). Second, by providing free online materials and not rigorously following any particular structure for responses, the ANIUF guarantees transparency among all participants (33).

Reviews of studies analyzing virtual communities of practice in healthcare have concluded that there is a paucity of reports on the methodology and process of maintaining these communities (15). This paper presents the first empirical analysis of ANUIF, evaluating participant recruitment, engagement, satisfaction, and solution generation across 26 months. In doing so, it contributes practical insights into building sustainable, scalable virtual innovation ecosystems in healthcare.

## 2 Materials and methods

### 2.1 Study design and participants

This observational, parallel mixed-methods study conducted a qualitative appraisal of 23 anonymous solutions generated by IF participants and a quantitative analysis of the ANUIF-generated solutions, participant numbers, and their level of satisfaction. Forum participants included ANU students and faculty involved in Alzheimer’s Disease and other Related Dementias (ADRD), as well as other researchers, physicians, nurses, social workers, medical assistants, certified nurse assistants, administrators, patients, their family members, advocates, and community health workers. Participation in the IF was voluntary; all participants were informed of the purpose of the IF, the documentation, and the distribution of the generated solutions, all of which were conducted under complete anonymity.

### 2.2 Innovation Forum procedures

**Figure 1:**
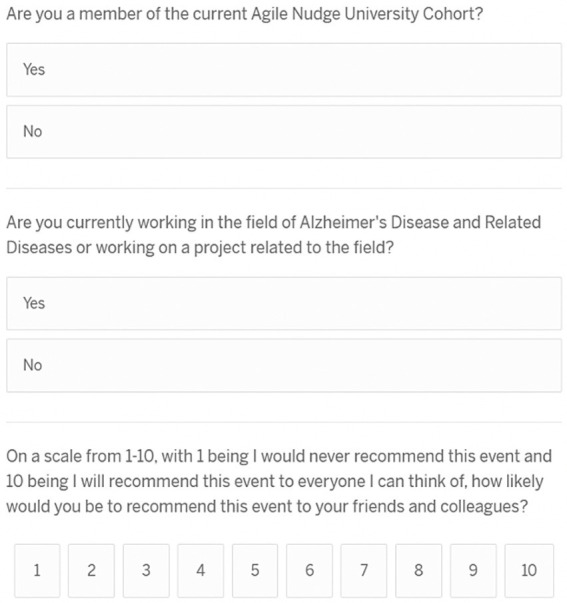
Innovation Forum evaluation survey. Example NPS calculation: The Net Promoter Score (NPS) is calculated as follows: first, the numbers and percentages of promoters (score of 9–10) and detractors (score of 0–6) are counted; second, the percentage of detractors is subtracted from the percentage of promoters. For example, if 10% of responses are from detractors (score of 0–6), 20% are passives (score of 7–8), and 70% are promoters (score of 9–10), the Net Promoter Score (NPS) would be 70–10 = 60.

**Figure 2:**
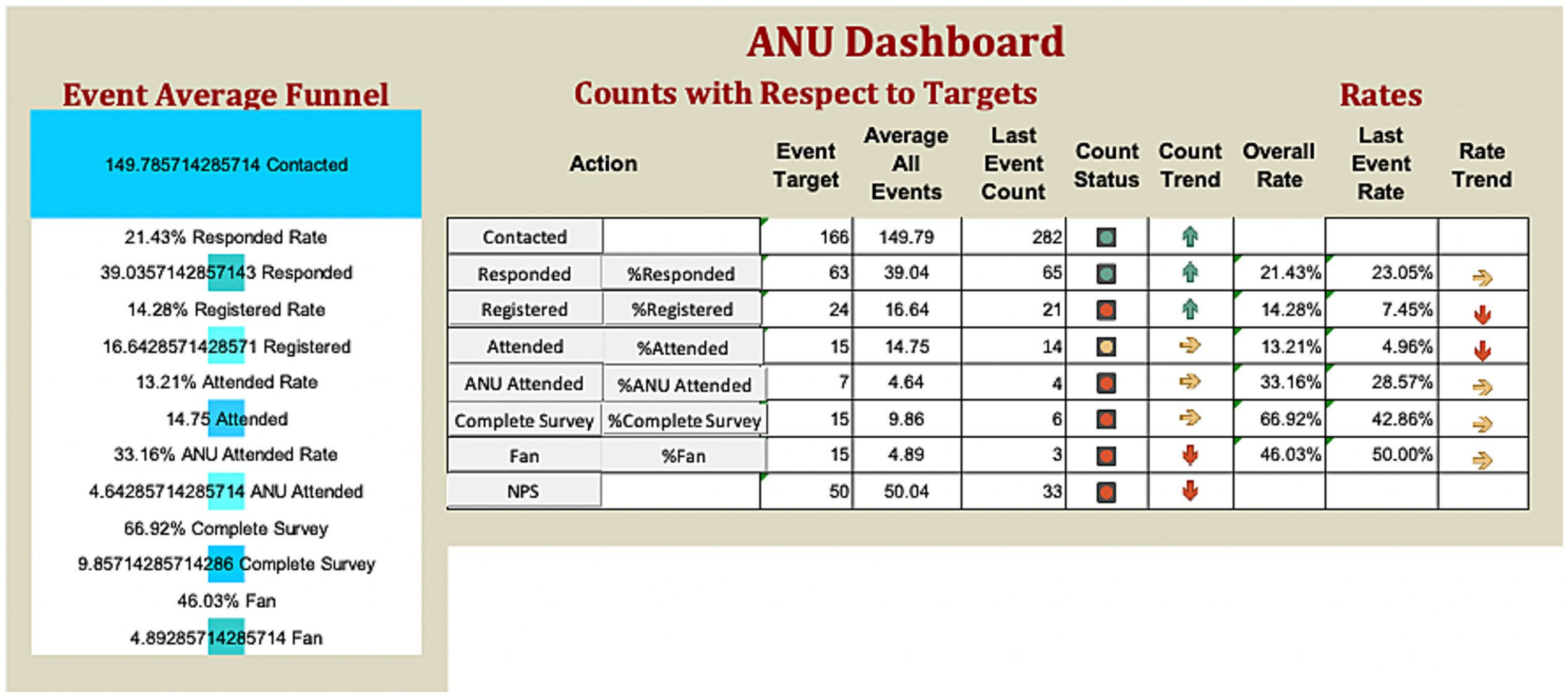
Agile Nudge University Innovation Forum dashboard. ANU, Agile Nudge University.

The ANUIF sessions are 90-min-long, monthly sessions with a consistent format, organized by a forum and administrative coordinators. During each forum, a different Presenter declared a “challenge” topic to the participating audience. Most IF presenters either held a Graduate Certificate in Health Innovation and Implementation Science or were graduates of the Agile Nudge University; thus, they had formal education in operationalizing nudges. The audience had 45 min to generate an original solution to the presented challenge, assuming no resource constraints; a tracker recorded the generated solutions, and a discussion facilitator enforced the ground rules of interaction to promote a positive environment. The solution tracker transcribed, recorded, and archived the generated solutions for each IF in a templated form. The administrative team, their roles, and the IF process are summarized in Table 1. The administrative coordinator ensured the availability of a challenge *Presenter* and the enrollment of at least 10 IF participants. 4 weeks before each forum, potential participants were contacted via email, and the presenter posted a challenge problem on a social media platform. 7–10 days before the event, reminders were sent to email respondents; some of the respondents registered and became attendees of the ANUIF. After each ANUIF, the administrative coordinator surveyed the participants’ satisfaction. Participants rated their likelihood of recommending the IF to other healthcare professionals on a scale from 0 to 10, and a Net Promoter Score (NPS) ranging from −100 to 100 was calculated (see Appendix for NPS calculation and Survey in Figure 1). Data from three cohorts of participants (Cohort 1 from 10/28/22–4/27/23, Cohort 2 from 5/22/23–4/11/24, and Cohort 3 from 5/30/24–12/30/2024) were collected from October 2022 until December 2024. The collected data, including the number of participants and NPS scores for each forum, were stored in Excel spreadsheets. In April 2024, an interactive dashboard was created to track and display the targeted number of participants contacted, respondents, registered, and attendees. The ANUIF interactive dashboard, featuring control charts (Figure 2), displayed traffic lights to signal deviations from target participant numbers at each stage of the process. A green and red arrow indicated that the average of the last four ANUIF attendees had been *two standard deviations* (SD) or more *above* and *below* the overall average, while a yellow arrow pointing to the right indicates that the average has been *within* 2 SD of the overall average. A cumulative funnel depicted attrition levels across different stages of recruitment, and tabs connected the dashboard to control charts for counts and rates. The control charts were color-coded according to the Nelson rules for detecting non-randomness (30). In April 2024, the NPS score was integrated into the ANUIF dashboard control charts.

**Table 1:** The Innovation Forum process and administrative team.

Activity	Time	Description
Opening networking	15 min	Attendees to introduce themselves and connect.
Presentation of the challenge	10 min	The presenter describes their implementation or delivery challenge using whatever visual aids they prefer.
Clarifying questions from the audience	5 min	The audience asks questions to clarify anything within the scope of the presentation. The Facilitator ensures no solutions are generated during this time and encourages each person to state his or her concern in question form.
Solution generation	45 min	Used for generating solutions, additional questions, and general brainstorming.
Closing discussion	15 min	Unstructured discussion to close.

Team member	Function
Forum coordinator	The primary organizer of the event; responsible for ongoing monitoring and evaluation of the event as well as maintaining communication with the presenter on any forum-related needs or preferences.
Presenter	Brings a challenge or problem to be solved to the event and may also identify a group of individuals and experts to whom personal invitations may be sent.
Facilitator	Conducts the Innovation Form, ensures smooth knowledge transfer between presenter and audience, and profiles and engages the audience. The Facilitator is not a content expert but promotes conversation, clarity, and understanding.
Solution tracker	Takes and distributes notes during Innovation Forum planning meetings and records solutions during the event day.
Administrative coordinator	Provides logistical and administrative support throughout the promotion and planning process and during the event.

### 2.3 Participants and forum composition

Each forum included participants with five types of expertise: (1) ANU Cohort members who recently completed a 3-day bootcamp on nudge and received ongoing mentoring; (2) individuals with prior training in nudge, such as CHIIS Graduate Certificate graduates and former ANU members; (3) nudge experts, including ANU faculty and experienced implementers across healthcare systems; (4) contextual experts from the healthcare system in which the intervention was being considered; and (5) subject-area experts, such as geriatricians when challenges focused on older adult care. This mix of perspectives ensured that solutions were based on both behavioral science and domain-specific knowledge.

### 2.4 Forum structure

Each forum was led by a facilitator who ensured that conversations stayed solution-focused rather than critical of past attempts. The challenge presentation and clarifying questions were given strict time limitations to avoid taking over the session and to allow enough time for the creation of solutions. Although facilitators occasionally used a nudge framework to generate ideas, participants brainstormed collaboratively most of the time without the aid of formal supports. This flexibility encouraged a wide variety of contributions and helped participants feel comfortable sharing creative solutions.

### 2.5 Innovation Forum data collection and variables

The variables analyzed include the number of contacts, respondents, registered individuals, forum attendees, and their NPS scores. Data were extracted from Excel spreadsheets from October 2022 until April 2024; data from April to December 2024 forums were extracted from the interactive dashboard.

### 2.6 Data analysis

#### 2.6.1 Qualitative data analysis

The ANUIF solutions, generated and recorded in templated forms, were accessed and de-identified. The files from each IF were merged and analyzed by a single researcher, with expertise in qualitative data analysis, using a combination of manual coding and NVivo software and applying thematic analysis to explore data systematically. We followed Braun and Clarke’s reflexive thematic analysis approach, using a combination of inductive and deductive coding strategies (31). As a single researcher conducted the coding, inter-rater reliability could not be established. However, by sharing the codebook with the rest of the research team members, group-related codes and connections between codes were cross-verified, and an iterative process of developing preliminary categories was initiated. The process included six steps: (1) familiarization with the data, (2) generation of initial codes (a mix of inductive codes emerging from the data and deductive codes informed by the study’s objectives and prior literature), (3) grouping of related codes to identify themes, (4) reviewing and refining themes, (5) defining and naming themes, and (6) producing a detailed report. In addition, thematic saturation was considered achieved after reviewing all 21 IF, as no new themes emerged during the final stages of analysis. To strengthen rigor, a reflexive approach was applied throughout the process. The coder maintained an audit trail documenting coding decisions, code definitions, and theme evolution to ensure transparency. Peer debriefing sessions were conducted with two senior researchers, who provided feedback on the coding framework and theme refinement, thereby reducing the influence of individual bias. Coding consistency was verified by revisiting earlier transcripts at multiple points during the analysis to confirm the stability of themes. Additionally, exemplary quotations were extracted for each theme to preserve participants’ voices and ground the findings in the raw data. These methodological safeguards collectively enhanced the credibility, transferability, and dependability of the qualitative results.

#### 2.6.2 Quantitative data analysis

Descriptive statistics, including cumulative counts, ranges, means, and SD, were calculated for each variable. Trends in the variable counts were graphically represented. Comparison of the variable counts (number of contacts, respondents, registrants, and attendees) before and after the implementation of administrative changes and the interaction dashboard, as well as the NPS for the three cohorts of attendees, was performed using *t*-test statistics. Correlation coefficients between the groups of contacts, respondents, registrants, and attendees were calculated using the Spearman correlation coefficient. An alpha level of 0.05 was used to determine significance. Statistical analyses were performed using Excel ToolPack. For each IF, the *solutions*, *sub-solutions* (specific parts of that solution, addressing a particular aspect of the problem), and *detailed solution*s (comprehensive explanations of the solution, including all necessary steps and reasoning) were tracked and recorded. Solutions were classified as “novel”- unique to the ANUIF, or *“existing solution”*- if previously generated in other forums and adapted to the challenge presented. The total number, averages, and SD were calculated for each solution category.

## 3 Results

### 3.1 Qualitative analysis

Qualitative analysis was performed for 21 IF; IF 22 and 23 data were unavailable. All sections of the forums were completed among the 21 IFs; therefore, since IFs 22 and 23 were not available, we excluded them from our data analysis process. Five main themes of the generated solutions emerged from the 21 IF analyzed:

- *Adaptive innovation* focused on flexibility and learning from change.
- *Collaborative Problem-Solving*, emphasizing teamwork and sharing knowledge to create solutions.
- *Data-driven improvement*, highlighting the use of feedback and evidence for continuous progress.
- *Practical and Relevant Solutions*, ensuring solutions are simple, user-centered, and applicable to real-world situations.
- *Sustainable and Scalable Impact*, focused on creating long-lasting, adaptable solutions. An example of a generated solution is presented in Table 2.

**Table 2:** Example of the generated solutions.

*Solution 1* Instead of depending on insurance companies, connecting with existing community groups already supporting this demographic would be highly advantageous. They could begin referring their clients for Vai’Datha services by forming partnerships with these groups. This approach would help expand her business by directly reaching those who benefit most from her expertise. Identifying areas with high demand and introducing herself to these communities as a consistent presence could encourage them to refer clients to her due to saliency bias. Being actively involved could make them valuable to their rehabilitation or program care plans. They would then meet the “simplest solution is the best” bias. Connect with hospital agencies and developmental clinics that are already doing this work and tap into their community. Perhaps also companies/businesses that are proactively building a neurodivergent workforce: Examples: Autism at Work (SAP), Autism Hiring Program (Microsoft), Neurodiversity@IBM, etc. Also, connecting with Autism Women & Nonbinary Network (AWN) awnnetwork.org; Autism Speaks, etc.
*Solution 2* Identify groups already providing educational resources or engaging young adults through an educational framework. Since these programs are often funded by federal dollars, explore opportunities to incorporate current work into their existing educational offerings. Using a social nudge, one could propose that services become a standard component of their educational plans.
*Solution 3* Implement a social nudge strategy by having different team members meet with groups that may have valuable connections (hospitals, clinics). Mapping out the social landscape of these services, identifying key connectors, and engaging with them can help establish the necessary relationships to advance her goals.

The following are examples of the identified themes that dominate the 21 IFs analyzed.

#### 3.1.1 Adaptive innovation

This theme emphasizes adaptability and receptivity to change. Solutions that accommodate user demands and promote learning from continuing encounters were proposed by participants.

- Implement a loss-aversion nudge: state the minimum requirement to maintain Agile Nudge University (ANU) membership; if not met, members will be eliminated from the tribe.
- Use a stoplight-style report (red, yellow, green) for Heart Rate Variability (HRV) data to encourage patients to adopt effective coping strategies.

#### 3.1.2 Collaborative problem-solving

The importance of collaboration and information exchange is shown in this theme. Working together to co-create successful strategies with peers, caregivers, and community members was emphasized in several solutions.

- Involve the patient’s partner in the caregiving process, ensuring someone in their care network is actively engaged.
- Utilize a deliberate messenger from the community who can reach people in a way that differs from a health provider to enhance the recruitment strategy.
- Introduce new ANU members to older cohorts across institutions, utilizing digital networking when in-person meetings are not possible.

#### 3.1.3 Data-driven improvement

This subject highlights how crucial it is to use feedback and proof. Participants frequently offered ideas for monitoring developments and using data to continuously improve solutions.

▪ Track the number of ANU applicants and calculate the completion percentage; if below 25%, terminate the application.
▪ Provide patients with a dashboard of HRV scores and associated events over time to identify stressors and evaluate coping strategies.

#### 3.1.4 Practical and relevant solutions

This topic emphasizes the importance of straightforward, practical, and user-centered concepts. The solutions were created to be easily accessible to the intended consumers and directly usable in daily practice.

▪ Establish telehealth visits from home to bridge mistrust and limited access.
▪ Ask invitees who decline Innovation Forums to provide reasons for not attending via the RSVP survey.
▪ Distribute Agile infographics and use precise vocabulary across the organization so values become part of daily practice.

#### 3.1.5 Sustainable and scalable impact

This theme focuses on developing solutions that are sustainable and go beyond a particular context. Participants concentrated on developing strategies that are flexible and accessible to a larger audience.

▪ Use asynchronous Innovation Forums over a day or a week to foster larger attendance and address time constraints.
▪ Expand superfans of Innovation Forums into trained facilitators, with ongoing monitoring and retraining as needed.

### 3.2 Quantitative analysis

**Figure 3:**
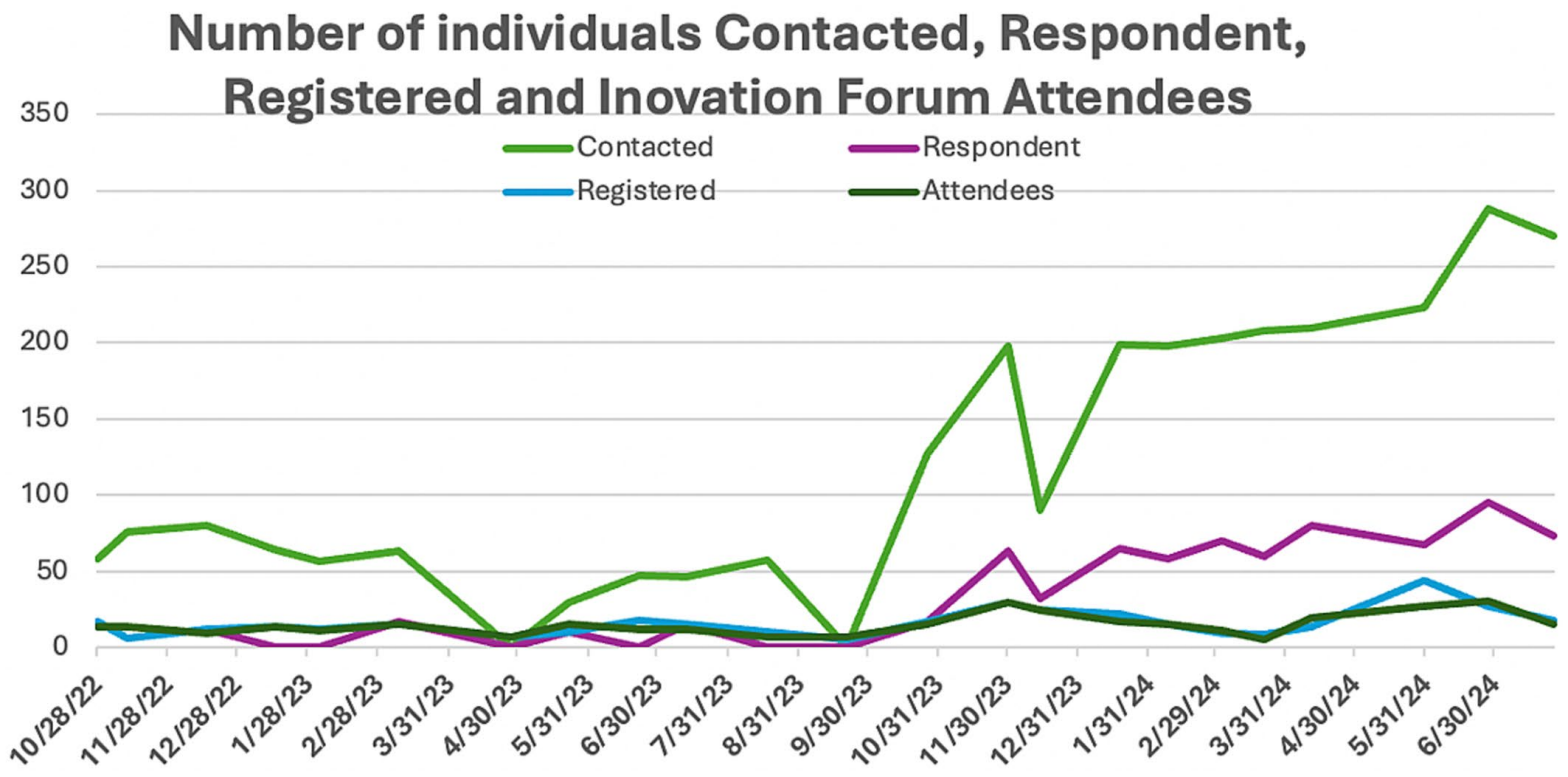
Trends in the numbers of contacted, respondents, registered individuals, and Innovation Forum attending participants.

**Figure 4:**
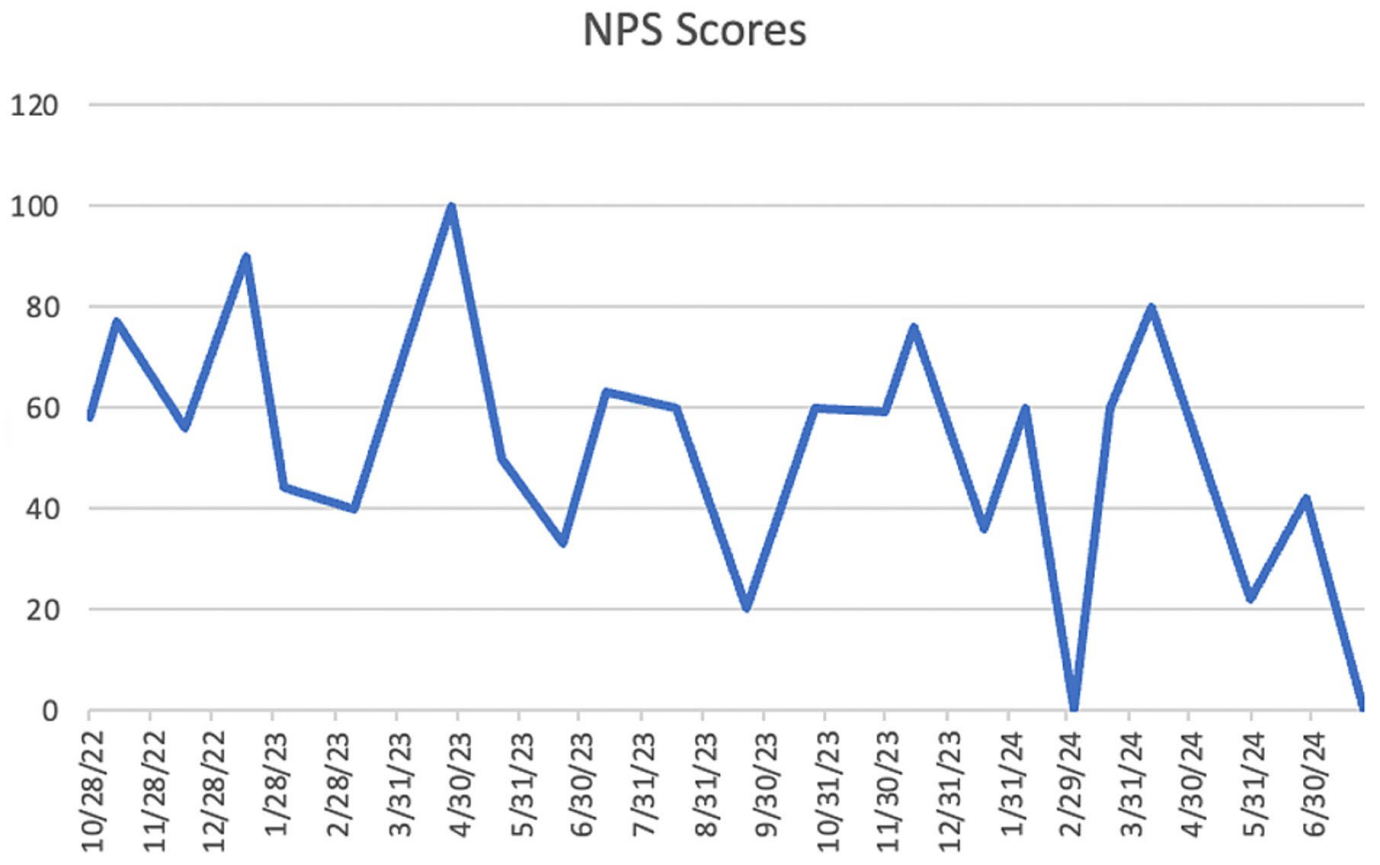
Innovation Forum attendees’ NPS (Net Promoter Scores).

**Figure 5:**
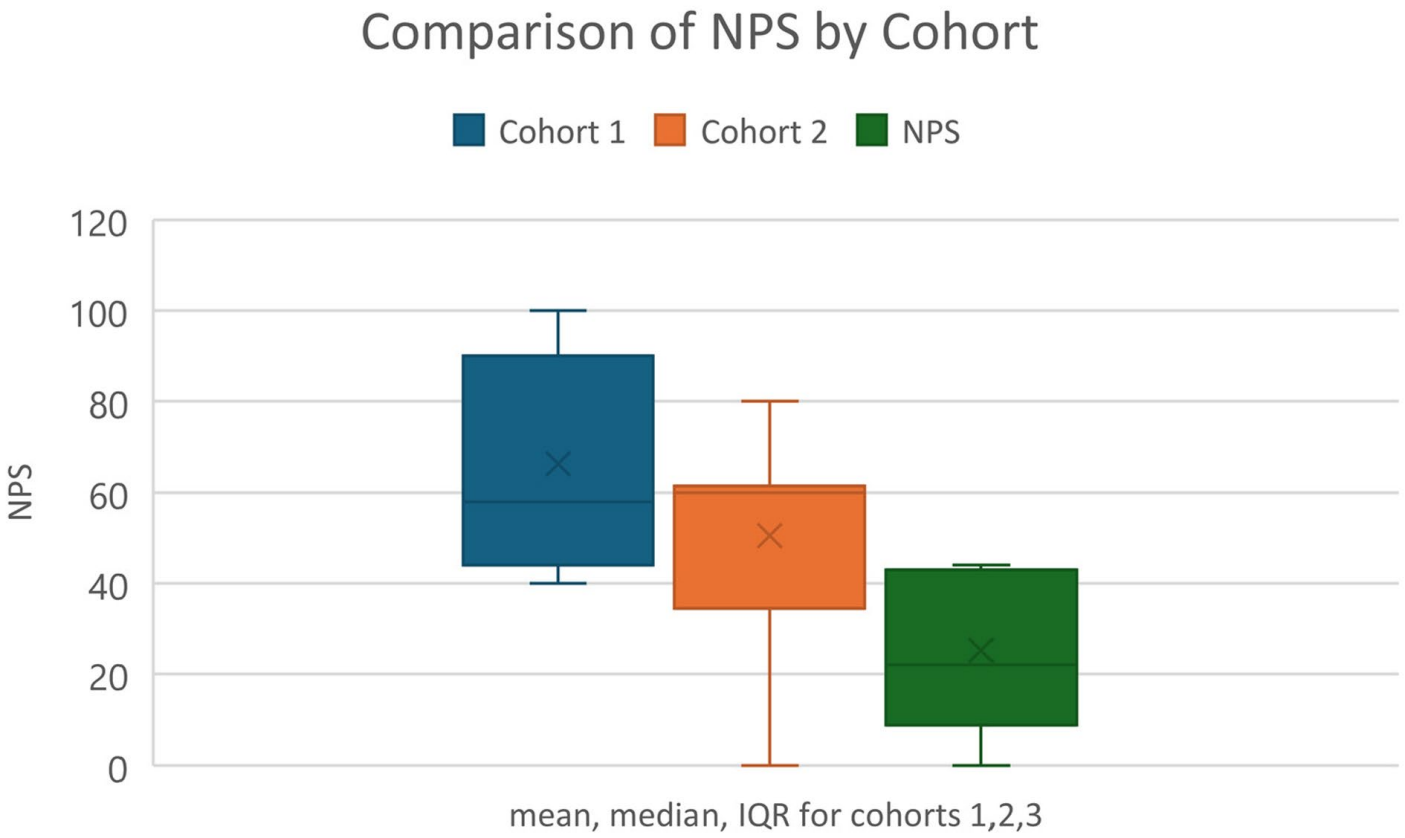
Boxplot representation of the NPS (Net Promoter Scores) by cohort of attendees.

(i) Table 3 presents the number of contacts, respondents, registered, and attending participants. 8.69% of the participant data was missing. The summary statistics, including ranges, means, and standard deviations, for contacted respondents, registered individuals, and attendees are presented in Table 4. Of the contacted individuals, 7.9–38% responded; 13–100% of the respondents registered for the event, and 61–100% of the registered participants, and occasionally more individuals, attended the forum. In five ANUIFs, the number of attendees was below 10. In Figure 3, line graphs represent the trends in the number of contracted, respondent, and registered IF participants during the study period. There were two notable spikes in the IF participants, one in November 2023 and another between May 2024 and June 2024, when the number of participants ranged from 27 to 30. The results of the comparison between the numbers of participants contacted, respondents, registered, and attendees before and after the introduction of administrative changes, along with the interactive dashboard control charts, are presented in Table 5. The correlation between the numbers of participants contacted, respondents, and registered is presented in Table 6. The post-ANUIF survey data were available for 21 of the 23 events, with a survey response rate ranging from 27 to 100%. Figure 4 presents the trends in the NPS scores of IF attendees. The mean NPS score for the 23 events was 51.56, SD = 25.08. The results of summary statistics and statistical testing of the differences in NPS scores between the three cohorts involved in the study are presented in Tables 7, 8, respectively. Boxplots of the NPS by cohort of attendees are shown in Figure 5.

**Table 3:** The counts of contacted, respondents, registered individuals and attendees; number of attendees who completed post Innovation Forum (IF) survey, NPS score.

Innovation Forum date	Number of individuals contacted	Number of individuals Respondent	Number of individuals registered	Number of attendees	Number of attendees who completed the Survey	NPS score
10/28/22	58	17	17	13	12	58
11/10/22	76	6	6	13	13	77
12/15/22	80	12	12	9	9	56
1/14/23	64	N/A	13	13	13	90
2/2/23	56	N/A	12	11	7	44
3/9/23	63	17	15	15	10	40
4/27/23	N/A	N/A	7	7	N/A	100
5/22/23	29	10	10	15	4	50
6/22/23	47	N/A	18	12	10	33
7/13/23	46	15	15	12	11	63
8/17/23	57	N/A	10	7	5	60
9/21/23	N/A	N/A	5	7	N/A	20
10/26/23	127	17	17	15	5	60
11/30/23	198	63	29	29	17	59
12/14/23	90	32	24	24	17	76
1/18/24	199	65	22	17	14	36
2/8/24	198	58	15	15	10	60
3/3/24	203	70	9	11	10	0
3/21/24	208	60	8	5	5	60
4/11/24	210	80	13	19	10	80
5/30/24	223	67	44	27	18	22
6/27/24	288	95	27	30	19	42
7/26/24	270	73	18	15	11	0

**Table 4:** Numbers, ranges, means, and standard deviation of contacted, respondent, registered individuals, and Innovation Forum (IF) attendees.

Descriptive statistics					
IF individuals	IF events with available data	Minimum number of individuals	Maximum number of individuals	Mean number of individuals	SD
CONTACTED	21	29	288	132.86	83.925
RESPONDENT	17	6	95	44.53	29.568
REGISTERED	23	5	44	15.91	8.852
ATENDEES	23	5	30	14.83	6.926

**Table 5:** Summary of statistical testing and significance of the difference between the number of contacts, respondents, registrants and attendees before-after IF dashboard implementation.

Groups	t-statistic	*p*-value	Cohen’s d	95% CI for difference in means
Contacts	8.27	0.000	3.54	(105.44, 181.9)
Respondents	7.27	0.000	3.14	(33.75, 63.03)
Registrants	2.64	0.010	1.12	(1.63, 16.13)
Attendees	3.02	0.005	1.28	(2.14, 13.16)

**Table 6:** Level of correlation between the counts of contacted, respondent, registered and attendees.

Group successions	Correlation between groups	95% CI
Contacts: respondents	0.9564	(0.90, 0.98)
Respondents: registrants	0.5049	(0.12, 0.76)
Registrants: attendees	0.8390	(0.65, 0.93)

**Table 7:** Net Promoter Scores (NPS) averages and standard deviations (SD) for the three attendee cohorts.

NPS score by cohort	Average	SD
Total	49.92	25.48
Cohort 1	66.43	23.01
Cohort 2	50.54	22.56
Cohort 3	25.20	18.25

**Table 8:** Statistical testing of Net Promoter Scores (NPS) comparison between attendee cohorts.

Cohort comparison	t-statistic	*p*-value	Cohen’s d	95% CI for difference in means
Cohorts 1–2	1.48	0.08	0.7	(−10.32, 42.1)
Cohorts 1–3	3.45	0.003	1.99	(8.11, 74.34)
Cohorts 2–3	2.46	0.017	1.23	(−3.22, 53.9)

(ii) The analysis included the categorization of solutions into four distinct types: main solutions, which represent the core strategies proposed; sub-solutions, which are smaller, complementary ideas that support or enhance the main solutions; detailed solutions, which involve more comprehensive, specific strategies; and total solutions, which combine all the proposed ideas, including both main and sub-solutions. The number of main solutions, sub-solutions, detailed solutions, total number of solutions, and existing and novel solutions for 21 Innovation Forums, averages, and SD are in Table 9. The average number of generated solutions per forum was 16 (SD = 5.1), with an average of 10.71 (SD = 3.16) *main solutions and* 4.38 (SD = 3.71) *sub-solutions.* The average number of old or *existing solutions* was 6.19 (SD = 2.94), and the average number of *novel solutions* was 4.52 (SD = 2.44).

**Table 9:** Numbers of main solutions, sub-solutions, detailed solutions, total number solutions, existing and novel solutions, for 21 Innovation Forums, averages and standard deviations (SD).

Innovation Forum	Main solutions	Sub-solutions	Detailed solutions	IF total number solutions	Existing solutions	Novel solutions
IF 1	6	10	0	16	5	1
IF 2	8	2	3	13	4	4
IF 3	8	2	0	10	5	3
IF 4	12	2	0	14	7	5
IF 5	10	5	2	17	6	4
IF 6	15	1	0	16	8	7
IF 7	13	0	0	13	13	0
IF 8	8	3	0	11	8	0
IF 9	17	6	6	29	12	5
IF 10	16	2	0	18	10	6
IF 11	9	4	2	15	7	2
IF 12	13	5	3	21	7	6
IF 13	13	0	0	13	6	7
IF 14	10	0	0	10	5	5
IF 15	10	6	0	16	7	3
IF 16	15	9	0	24	7	8
IF 17	6	4	1	11	3	3
IF 18	9	9	0	18	2	7
IF 19	8	13	0	21	4	4
IF 20	11	9	2	22	2	9
IF 21	8	0	0	8	2	6
Average	10.71	4.38	0.90	16.00	6.19	4.52
SD	3.16	3.71	1.54	5.10	2.94	2.44

## 4 Discussion

**Figure 6:**
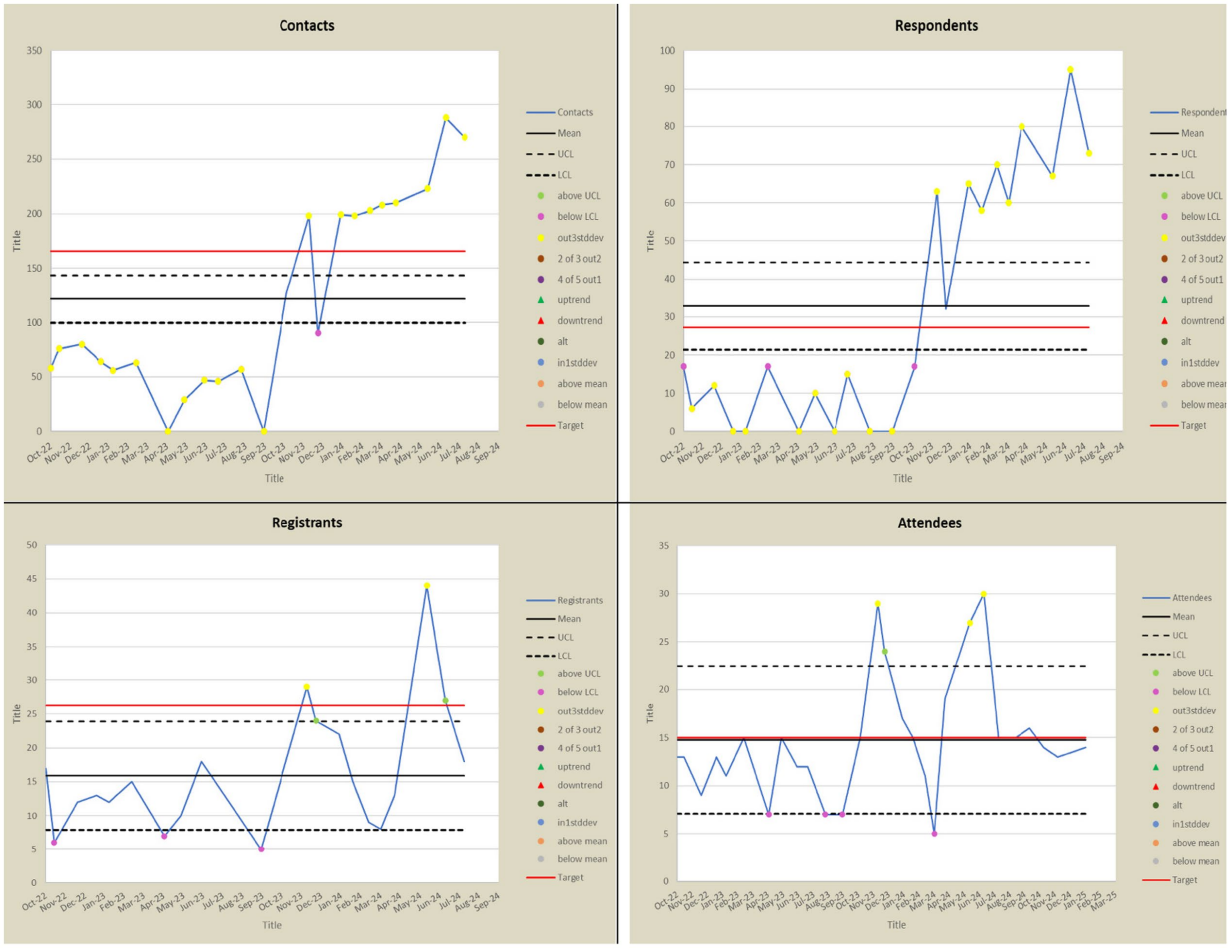
Control charts with the trends in the numbers of Contacts, Respondents, Registrants, and Attendees. UCL, Upper control Limit; LCL, Lower Control Limit.

The ANUIF environment values innovation, creative thinking, and evidence-based problem-solving, leveraging agile science methodology. Its success depends on streamlined recruitment processes that ensure a minimum number of participants. While the average number of IF *attendees* was 15, the number went up to 30. Although the number of respondents and registered participants varied throughout the study, the number of attendees increased in the latter forums. While these participation measures varied, this was understandable given the ANUIFs’ real-world, interdisciplinary nature. Importantly, the data indicated relevant patterns, such as continuous gains after administrative adjustments and improved solution creation across forums. The low attendance shown on the control charts during 2022 and early 2023 prompted a reorganization of the administrative team and a reassignment of their responsibilities in September 2023. In October 2023, there was a sharp increase in the number of *contacted* and *respondent* individuals, mirrored by a rise in the number of *registered* individuals and *attendees* visible on all the control charts in Figure 6. The administrative changes and implementation of the ANUIF dashboard led to a statistically significant increase in the number of contacts, respondents, registrants, and attendees, as summarized in Table 5. The correlations between the successive counts of contacted, respondent, registered, and attendees are summarized in Table 6. Investigations into the causes of low r*egistrants* and *attendees* in March 2024 revealed two key factors. First, busy healthcare professionals are unable to participate in two ANUIFs in the same month. Second, the speaker in late March 2024 was unknown to ANUIF students and regular attendees. In May 2024, the control chart displayed a decline in NPS (Figure 4). The administrative team has determined that the response rate to the Survey declined with successive ANU cohorts (Figure 5) and that NPS changes were unrelated to the number of attendees. The differences in scores between cohorts were statistically significant, and the causes of decline in NPS were further investigated. To determine the cause of the NPS trends, the administrative team is testing the format of the ANUIF-generated solutions.

Implementing administrative changes and the interactive dashboard improved recruitment tracking and streamlined the processes. By alerting event stakeholders to any deviations from target participant numbers at different stages of the organizational process, the dashboard’s traffic lights, and trend arrows signaled a need for active intervention. Integrating the NPS on the ANUIF dashboard allowed fluent score analysis and interpretation. Despite the research period’s NPS average of 51, the scores were heading downward. Although there were minor variations in NPS between cohorts, the consistently high average points to generally satisfying participant experiences. Rather than a general lack of satisfaction, the variety can be the result of variations in the facilitation, the content, or the backgrounds of the participants.

The ANUIF themes of the generated solutions proved generalizable and applicable to “real-world” healthcare challenges, enforcing the ANUIF’s function of “built-in consulting.” In all 21 forums analyzed, participants generated more than one solution and often several sub-solutions to the problems discussed, demonstrating an understanding of problem-solving, ideation, and innovation. Once generated, solutions were narrowed down to candidate solutions based on predefined criteria. As previously described, the convergence or single solution selection is based on scientific evidence and prioritizes investment in the most promising ideas (17). The solution generators combined or modified elements of *existing solutions* in novel ways to apply and localize them to new problems or delivered *novel solutions* previously unknown to the IF. These approaches are not new and have been identified in the behaviors of successful innovators (5). ANUIF participants have demonstrated the ability to be critical during the idea-generation process; their experiences, journeys, needs, and preferences contributed uniquely and significantly to finding a solution (19, 20).

While organizing the IF events is feasible and the planning, invitation, and facilitation can be done by a single individual, scaling the generated solutions across healthcare systems presents several challenges. Although the forums successfully generated diverse and often practical solutions, scaling these ideas across healthcare systems presents several challenges. First, many solutions were context-specific, rooted in local institutional culture, available resources, and participant expertise; thus, limiting their direct applicability to other healthcare settings. Additionally, nudges are by design small behavioral interventions, but to move from ideation to widespread adoption requires robust institutional support, leadership buy-in, and adaptation to existing workflows. Without dedicated implementation pathways and accountability mechanisms, promising ideas risk remaining “pilot solutions” that fail to achieve broader health system change.

Participants’ time constraints limited both attendance at forums and engagement in follow-up implementation activities. Moreover, while the dashboard streamlined recruitment and tracking, it did not fully address deeper systemic issues, such as ongoing facilitation beyond the initial ideation stage. An important observation was that not all forums generated nudge-focused solutions; instead, several forums produced broader strategies. This gap may reflect the complexity of specific healthcare challenges, where behavioral nudges alone are insufficient to address them, or there is a lack of evidence linking the proposed behavior change to measurable outcomes. This scientific rigor, while appropriate, sometimes limited the generation of quick, practical nudges, leading instead to more traditional problem-solving approaches.

The lack of nudge orientation in some cases may also reflect the diversity of forum participants. While some individuals had formal training in behavioral science and agile innovation, many other members generated solutions framed in operational or systemic terms. Future forums may benefit from integrating nudge-specific prompts or training modules into the solution-generation process to ensure that behavioral strategies are consistently considered alongside broader innovations. Taken together, these findings suggest that while the ANUIF has proven effective in fostering collaborative, cross-disciplinary innovation, its scalability is dependent on stronger infrastructure for diffusion, more precise alignment with evidence-based nudge design, and strategies to overcome systemic barriers that can dilute or block promising solutions from moving beyond the forum stage.

### 4.1 Forum structure and contribution to the field

The forum structure itself contributed to both successes and challenges. Facilitators and explicit time limits were crucial to preserving the focus and productivity of the sessions. Furthermore, the platform became more accessible to professionals in their early and mid-career stages by bringing in specialists from a variety of disciplines, which increased the caliber of the solutions produced and expanded their networks. But sometimes, the absence of a structured methodology, such as design thinking, or a formal nudge framework led to solutions that went beyond behavioral nudges. However, by bridging gaps between specialties and levels of expertise, the forums were beneficial to the profession. In order to support speakers and encourage interdisciplinary collaboration, they established a strong and connected network of specialists, which served as the cornerstone for more all-encompassing healthcare innovation.

### 4.2 Online collaboration and sustainability

Collaboration and innovation on online platforms can transform the healthcare industry. Online communities have been shown to enhance knowledge and improve practice (32). Like other thriving online communities (28), the ANUIF focuses on developing evidence-based nudges to address real-world problems. It has a well-defined structure and process and fosters a sense of community. Sustaining it and managing its growth, activity, and design are iterative processes that must adapt to the needs of its members and the community’s purpose (29, 30).

## 5 Limitations

Our paper has several limitations. First, although the Innovation Forum’s original purpose was to design, implement, or assist with the diffusion of a nudge, not all solution groups produced nudge strategies for patients or clinicians. This lack of nudge-focused outputs often stemmed from the complexity of the presented problems, which could not be addressed solely by behavioral adjustments, or from participants’ caution in proposing nudges without a strong evidence base. Second, since the participants of the ANUIF practice in broad geographic areas, this project lacks feedback on the real-world implementation and impact of proposed solutions. Third, the generalizability of findings is limited. The forums drew participants primarily from healthcare professionals, researchers, and advocates affiliated with a specific academic network, which may not fully represent the diversity of healthcare systems or patient communities. Solutions generated may therefore reflect biases of the participant pool and may not translate seamlessly to other settings. Fourth, missing data presented challenges for analysis.

In some cases, participant response rates were incomplete, survey data were not returned, and solution documentation was inconsistent. While statistical adjustments were made where possible, gaps may have influenced both the quantitative results and the qualitative interpretation. Finally, the outcome measures used, such as Net Promoter Score (NPS) and participant satisfaction capture perceptions of the forum experience, but not the downstream effectiveness of solutions in real-world practice.

## 6 Conclusion

The ANUIF is a unique channel for open communication, creation, and knowledge sharing. It fosters cross-functional collaboration and the exchange of ideas among stakeholders, leading to innovation. The diversity of problems the ANUIF addresses, along with the number of solutions generated, demonstrates the forum’s flexibility and versatility, as well as the participants’ wealth of knowledge and ability to generate sustainable, user-centered innovations that solve real-world problems.

The study’s findings include steady participation rates following administrative restructuring, measurable improvements in recruitment tracking through the dashboard, the generation of multiple solutions per forum, and the identification of five consistent qualitative themes. These results confirm that the ANUIF process can reliably engage participants and stimulate the generation of diverse solutions.

In contrast, some outcomes remain aspirational. While forums successfully produced innovative ideas, the study did not assess whether these solutions were adopted, sustained, or effective in improving patient or system-level outcomes. Broader scalability, long-term implementation, and measurable healthcare impact are goals for future research rather than confirmed achievements. Structured follow-up mechanisms, collaborations with implementation partners, and objective outcome measures will be necessary to transform the promise of ANUIF solutions into demonstrated real-world impact.

## Data Availability

The raw data supporting the conclusions of this article will be made available by the authors, without undue reservation.
